# Pulmonary Screening Practices of Otolaryngology–Head and Neck Surgeons Across Saudi Arabia in the Posttreatment Surveillance of Squamous Cell Carcinoma: Cross-sectional Survey Study

**DOI:** 10.2196/24592

**Published:** 2022-03-18

**Authors:** Majed Alnefaie, Abdullah Alamri, Asalh Saeedi, Awwadh Althobaiti, Shahad Alosaimi, Yousuf Alqurashi, Hani Marzouki, Mazin Merdad

**Affiliations:** 1 King Fahad Armed Forces Hospital Medical Services of The Armed Forces Jeddah Saudi Arabia; 2 Department of Otolaryngology Head and Neck Surgery Faculty of Medicine King Abdulaziz University Jeddah Saudi Arabia

**Keywords:** squamous cell carcinoma of head and neck, lung neoplasms, radiography, otolaryngology, surgeons, survey

## Abstract

**Background:**

With respect to patients with head and neck squamous cell carcinoma (HNSCC), posttreatment surveillance for distant disease has mostly focused on the lungs, as HNSCC distant metastasis occurs in this organ in 90% of HNSCC cases. Additionally, the incidence rate of primary tumors in the lungs is high due to the field cancerization of the entire upper aerodigestive tract.

**Objective:**

Our cross-sectional survey study aims to evaluate the current beliefs and pulmonary screening practices of otolaryngology–head and neck surgeons across Saudi Arabia with respect to the posttreatment surveillance of HNSCC.

**Methods:**

This nationwide cross-sectional survey was conducted among head and neck surgeon members of the Saudi Society of Otolaryngology from June 1 to June 30, 2020. A predesigned questionnaire was used for data collection, and a descriptive analysis was carried out.

**Results:**

This study included 22 participants and had a 78% (22/28) response rate. This study found that the majority of participants (9/22, 41%) used lung radiography for routine lung screening during posttreatment follow-ups, whereas 32% (7/22) used low-dose computed tomography (CT; 7/22, 32%). With regard to the number of years for which participants perform lung screening during follow-ups, the majority of participants (17/22, 77%) reported 5 years, and only 9% (2/22) have performed lifelong lung screening. With regard to the frequency of lung screening, 77% (17/22) of participants conduct screening annually, 18% (4/22) conduct screening half-yearly, and 5% (1/22) conduct screening biennially. With regard to beliefs about the effectiveness of screening procedures in reducing lung cancer mortality rates during follow-ups, 36% (8/22) of participants believed them to be very effective or somewhat effective, 18% (4/22) did not know, and only 9% (2/22) believed that they were not effective.

**Conclusions:**

The participants mainly used lung radiography (9/22, 41%), low-dose CT (7/22, 32%), or positron emission tomography/CT (6/22, 27%) as a routine lung screening method during the posttreatment follow-up of patients with head and neck cancer for 5 years (17/22, 77%) or 10 years (3/22, 14%), and only a small percentage of participants have performed lifelong lung screening (2/22, 9%). Lung screening was mostly conducted annually or half-yearly. Such screening was believed to be very effective or somewhat effective.

## Introduction

Distant head and neck squamous cell carcinoma (HNSCC) metastases are discovered at various sites, but they most often occur in the lungs (66%-83% of HNSCC cases) and commonly occur in bones (22%-31% of HNSCC cases) and in the liver (6%-10% of HNSCC cases) [1]. The reported prevalence rates of HNSCC metastases in different clinical studies vary from 4% to 26%, and those reported in postmortem research vary from 37% to 57% [2].

There are many variables that impact the growth of distant metastases, such as the primary site, histological differentiation, patients’ immunological abilities, advanced tumor stages, locoregional primary tumor control, and the extracapsular metastasis of the lymph nodes [3].

Metastasis is the natural evolution of primary tumors in patients with advanced HNSCCs who are not undergoing locoregional primary tumor control. Both distant metastases and secondary primary carcinomas may grow over time in curatively treated patients. This patient group may benefit from undergoing follow-ups after receiving therapy for the main HNSCC if the secondary cancer has been cured [4].

Screening for distant metastases and secondary primary tumors in the lungs is helpful, as it allows clinicians to make prognoses and allows for adapted patient counseling. Further, such screening has a beneficial effect on the prognoses of patients when it results in the early detection of distant metastases and secondary primary tumors [5].

Pulmonary follow-ups for secondary lesion identification can be performed in many ways, including via chest x-rays, computed tomography (CT) scans, positron emission tomography (PET) scans, bronchoscopy, brushes, and cytology [6].

A relatively recent field of research is the design of follow-up programs. Posttreatment follow-ups have been acquiring importance in clinical settings. The optimal form of surveillance is not clear, and there is a lack of data on the cost-effectiveness of rigorous monitoring. Despite the different timing protocols and the different modalities that are used among clinicians, the common denominator is the objective of promptly detecting and treating recurrent diseases as well as secondary primaries. Nowadays, multi-professional teams with skills in treatment toxicity management and prevention conduct follow-ups. The current follow-up methods include full head and neck clinical examination and structural examination [7-9].

In a previous study that was conducted by otolaryngology–head and neck surgeons, 26 out of 32 participants performed routine pulmonary screening, and of these 26 participants, 23 (88%) believed that chest radiography should be the preferred screening method. Most participants thought that mortality could be beneficially affected by lung screening. The most preferred modality for screening symptomatic patients was low-dose spiral CT (48%), followed by PET/CT (14%) and sputum cytology (14%). Additionally, 31% of respondents performed a chest x-ray for high-risk asymptomatic patients (current smokers, patients exposed to radiation, patients with a family history of cancer, and patients with advanced HNSCC). The same percentage of respondents conducted low-dose CT, while 19% relied on PET scans. Further, 19% of respondents did not screen any high-risk patients. Most respondents (77%) had more than 10 years of medical practice since graduating from medical school in the provinces of Quebec, Ontario, and Alberta [10].

Our cross-sectional survey study aimed to evaluate the current beliefs and pulmonary screening practices of otolaryngology–head and neck surgeons across Saudi Arabia with respect to the posttreatment surveillance of HNSCC. In this study, the findings of our survey were compared to the most recent data from the literature.

## Methods

### Study Design, Duration, and Participants

We used an analytical cross-sectional study design and collected data during the period from June 1 to June 30, 2020. Head and neck surgeon members of the Saudi Society of Otolaryngology in Saudi Arabia were surveyed in this study.

### Inclusion and Exclusion Criteria

All head and neck surgeons of the Saudi Society of Otolaryngology who worked in Saudi Arabia hospitals and consented to participate in this study were included. There were no exclusion criteria.

### Sample Size

The total sample consisted of all head and neck surgeons of the Saudi Society of Otolaryngology in Saudi Arabia. A total of 22 participants were included in this study.

### Study Procedures

We adapted a questionnaire that consisted of 6 questions regarding actual practices and was previously designed and reviewed by Madana et al [10]. The questionnaire was used after obtaining permission from the main author. The questions inquired about the characteristics of routine lung screening during the posttreatment follow-up of patients with head and neck cancer [10]. The questionnaire was distributed to all head and neck surgeons of the Saudi Society of Otolaryngology in Saudi Arabia. No translation was needed, as the distributed form was written in the English language.

### Data Management and Statistical Analysis

We used SPSS version 26 (IBM Corporation) to analyze the study data. Descriptive statistics were used to present the frequencies and percentages of the categorical variables.

### Ethical Considerations

We prepared an informed consent form for the participants and gave them a brief description of this study’s rationale and objectives. Afterward, we asked them to sign the consent form. The anonymity and confidentiality of data were maintained throughout the study. Records were retained in a password-protected computer, and they will be retained for at least 7 years. There were no conflicts of interest. The study was approved by the Unit of Biomedical Ethics Research Committee at King Abdulaziz University.

## Results

With regard to the methods of routine lung screening that were used during the posttreatment follow-up of patients with head and neck cancer, our study found that the majority of participants (9/22, 41%) used lung radiography, whereas 32% (7/22) used low-dose CT and 27% (6/22) used PET/CT. With regard to the number of years for which physicians perform lung screening for head and neck cancer during follow-ups, the majority of participants (17/22, 77%) reported 5 years and 14% (3/22) reported 10 years; only 9% (2/22) have performed lifelong lung screening. With regard to the frequency of lung screening, 77% (17/22) of participants conduct screening annually, 18% (4/22) conduct screening half-yearly, and 5% (1/22) conduct screening biennially. With regard to the believed effectiveness of the screening procedures (ie, those listed in question 1) in reducing lung cancer mortality rates during the follow-up of patients with head and neck cancer, 36% (8/22) of participants believed them to be very effective or somewhat effective, 18% (4/22) did not know, and only 9% (2/22) believed that they were not effective (Table 1).

**Table 1:** Characteristics of routine lung screening during the posttreatment follow-up of patients with head and neck cancer (respondents: N=22).

Parameters		Respondents, n (%)
**Methods of routine lung screening during the posttreatment follow-up of patients with head and neck cancer**		
	Low-dose computed tomography	7 (32)
	Lung radiography	9 (41)
	Positron emission tomography/computed tomography	6 (27)
**Types of patients with head and neck cancer who underwent routine lung screening during posttreatment follow-ups**		
	All patients	9 (41)
	Only high-risk patients (smokers, patients exposed to radiation, patients with a family history of cancer, and patients with advanced HNSCC^a^)	9 (41)
	Only symptomatic patients	4 (18)
**Number of years for which physicians perform lung screening for head and neck cancer during follow-ups**		
	10 years	3 (14)
	5 years	17 (77)
	Lifelong	2 (9)
**Physicians’ frequency of conducting lung screening for head and neck cancer during follow-ups**		
	Annually	17 (77)
	Biennially	1 (5)
	Half-yearly	4 (18)
**Believed effectiveness of the screening procedures (ie, those listed in question 1) in reducing lung cancer mortality rates during the follow-up of patients with head and neck cancer**		
	Did not know	4 (18)
	Not effective	2 (9)
	Somewhat effective	8 (36)
	Very effective	8 (36)
**Have any of the patients during the past 12 months inquired about lung screening?**		
	No	11 (50)
	Yes	11 (50)
**Number of years of clinical head and neck practice and number of years since graduation from medical school**		
	0-5	1 (5)
	11-20	9 (41)
	6-10	6 (27)
	>20	6 (27)
**Practicing census region**		
	Asir	1 (5)
	Dammam	2 (9)
	Jeddah	8 (36)
	Jazan	1 (5)
	Mecca	2 (9)
	Riyadh	7 (32)
	Ta'if	1 (5)
**Patient volume during a typical week of head and neck practice (number of patients/week)**		
	20-50	11 (50)
	50-75	3 (14)
	75-100	1 (5)
	<20	7 (32)

^a^HNSCC: head and neck squamous cell carcinoma.

## Discussion

### Principal Findings

A high response rate among otolaryngology–head and neck surgeons across Saudi Arabia was achieved in our study (22/28, 78%). This shows a high level of interest in postoperative screening practices.

Head and neck cancer refers to a group of malignant neoplastic lesions that have similar biological behaviors and are found in the upper aerodigestive tract. Head and neck cancer is the sixth most common cancer in the world; each year, over 500,000 new cases are diagnosed and 200,000 related deaths occur [11,12]. The most common sites of distant metastases are the lungs, the skeletal system, and the liver [13]. Due to the high incidence rate of metastasis (90% of cases) in patients with HNSCC, the posttreatment examination of the pulmonary region is critical [14,15]. Patients with HNSCC need posttreatment care that does not end with the completion of definitive treatment.

Our nationwide survey was conducted among head and neck surgeon members of the Saudi Society of Otolaryngology. The purpose of this survey study was to assess otolaryngology–head and neck surgeons’ current beliefs and pulmonary screening practices with respect to the posttreatment surveillance of HNSCC in Saudi Arabia.

Regrettably, there is no consensus in the literature on the frequency and mode of posttreatment follow-up. Different investigational modalities each have their own set of advantages and disadvantages [16].

Similar to our results, another study, which was conducted by the Canadian Society of Otolaryngology to evaluate head and neck surgeons, reported that the majority of respondents performed routine lung screening and preferred chest radiography over low-dose CT or PET [10]. There is evidence however that PET/CT may be the most sensitive of these modalities, but further research is needed to show improvements in patient outcomes [16]. According to the Centers for Disease Control and Prevention, the most recommended method for lung cancer screening is low-dose CT [17]. Additionally, the present guidelines of the US Preventive Services Task Force suggest using the same method [18]. The reason why physicians prefer to avoid low-dose CT in follow-ups that are conducted after the treatment of head and neck cancer may be the modality’s low specificity. The overdiagnosis of lung cancer was reported in more than 18% of cancer cases during the screening process of the National Lung Screening Trial [19]. Depending on radiography however cannot be the correct decision to make since, in another study, radiography was proven to be a poor method for diagnosing lung tumors in more than 65% of patients with cancer, and these patients were later diagnosed with pulmonary cancer [20]. Thus, the most recent findings among physicians must be disseminated more frequently.

With regard to the number of years for which participants perform lung screening during follow-ups, the majority of participants (17/22, 77%) reported 5 years, and only 9% (2/22) have performed lifelong lung screening. The study conducted in Canada found that 60% of their respondents conduct lung screening for 5 years, some of their respondents conduct lifelong lung screening, and the fewest number of participants conduct lung screening for 10 years [10]. This difference in the number of years for which physicians perform lung screening can be attributed to the variations in the current evidence concerning the posttreatment follow-up of patients with head and neck cancer; some studies have suggested that physicians should continue to conduct follow-ups once per year after 5 posttreatment years [21]. However, there is little evidence that supports the effectiveness of conducting follow-ups for more than 5 years [22].

The British Association of Head and Neck Oncologists [23] recommends 4- to 6-week follow-up visits for the first 2 years after treatment, 3-month follow-up visits for the third posttreatment year, 6-month follow-up visits for the fourth and fifth posttreatment years, and annual visits after that. With regard to the frequency of lung screening, in our study, the majority of participants (17/22, 77%) conduct screening annually, 18% (4/22) conduct screening half-yearly, and 5% (1/22) conduct screening biennially.

The Canadian study reported that most respondents were screening their patients annually, while less than 15% screened patients biennially or half-yearly [10]. Variations in the number of follow-up visits have also been evident in the guidelines present in the literature and can account for the differences between Canada and Saudi Arabia, since clinicians from different countries can follow different guidelines [9]. The Saudi head and neck surgeons in our study stated that conducting scheduled visits is also the best way to provide adequate follow-ups to patients with HNSCC, since these follow-ups address many concerns and not just the early detection of recurrence or secondary primaries.

### Strengths and Limitations

To date, no study has been conducted in Saudi Arabia to analyze the current practices of head and neck surgeons with respect to detecting post-HNSCC pulmonary metastasis. Our study provides a highly comprehensive view of current practices, given that all certified head and neck surgeons of the Saudi Society of Otolaryngology in Saudi Arabia participated in this study. Despite its exploratory nature, this study offers some insight into the lack of evidence-based practices for the posttreatment pulmonary surveillance of HNSCC. Conducting well-controlled trials to evaluate different modalities of surveillance for different subtypes and stages of HNSCC would shed light on the survival rates associated with and the cost-effectiveness of these modalities. However, we are limited by the cross-sectional nature of this study. We are also limited by the lack of literature on this topic and, hence, our inability to obtain enough confidence in our results.

### Conclusion and Recommendations

The participants mainly used lung radiography (9/22, 41%), low-dose CT (7/22, 32%), or PET/CT (6/22, 27%) as a routine lung screening method during the posttreatment follow-up of patients with head and neck cancer for 5 years (17/22, 77%) or 10 years (3/22, 14%), and only a small percentage of participants have performed lifelong lung screening (2/22, 9%). Lung screening was mostly conducted annually or half-yearly. Such screening was believed to be very effective or somewhat effective. However, controversy still exists due to the lack of evidence-based protocols worldwide. Therefore, future research should explore the importance of this subject by using a more comprehensive methodology and enrolling patients with HNSCC in comparative studies. We also recommend conducting further follow-up studies to obtain more knowledge on the effects of the positions that physicians hold.

## Abbreviations

CT: computed tomography
HNSCC: head and neck squamous cell carcinoma
PET: positron emission tomography
